# Development of a liquid biopsy based purely quantitative digital droplet PCR assay for detection of *MLH1* promoter methylation in colorectal cancer patients

**DOI:** 10.1186/s12885-021-08497-x

**Published:** 2021-07-10

**Authors:** Danyi Wang, Dennis O’Rourke, Jorge F. Sanchez-Garcia, Ti Cai, Juergen Scheuenpflug, Zheng Feng

**Affiliations:** 1 Global Clinical Biomarkers and Companion Diagnostics, Global Early Development, EMD Serono Research and Development Institute, Billerica, MA, USA; 2 Global Clinical Biomarkers and Companion Diagnostics, Global Early Development, Merck Biopharma, Merck KGaA, Darmstadt, Germany

**Keywords:** *MutL homolog 1*, Circulating tumor DNA, Methylation, Methylation sensitive restriction enzyme, Droplet digital PCR

## Abstract

**Background:**

*MutL Homolog 1* (*MLH1*) promotor methylation is associated with microsatellite instability high colorectal cancer (CRC). The strong correlation between methylation status and cancer development and progression has led to a growing interest in the use of methylation markers in circulating tumor DNA (ctDNA) for early cancer detection and longitudinal monitoring. As cancer-specific DNA methylation changes in body fluids are limited, it is particularly challenging to develop clinically applicable liquid biopsy methodologies with high sensitivity and specificity. The purpose of this study was to develop a fit-for-purpose methylation sensitive restriction enzyme (MSRE) based digital droplet PCR (ddPCR) assay to examine *MLH1* promoter methylation in ctDNA in advanced CRC.

**Methods:**

Primers and probes were designed to amplify CpG sites of the *MLH1* promoter. Methylated and unmethylated control genomic DNA were sheared to mimic ctDNA and subjected to MSRE *HpaII* digestion. Plasma samples from 20 healthy donors and 28 CRC patients were analyzed with the optimized MSRE procedure using ddPCR.

**Results:**

Using methylated and unmethylated controls, we optimized the conditions for *HpaII* enzyme digestion to ensure complete digestion and avoid false positives. Based on the results from the ddPCR assay using 1 ng circulating cell-free DNA (cfDNA) input from healthy donors or CRC samples, ROC curves were generated with an area under the curve (AUC) value of 0.965 (95% CI: 0.94, 0.99). The statistically optimal assay sensitivity and specificity was achieved when 8 positive droplets were used as acceptance criteria (78% sensitivity and 100% specificity, 95% CI: 0.45, 0.95). A tiered-based cutoff (20, 50, 80% percentile based) was applied to distinguish CRC samples with different methylation level.

**Conclusions:**

Our study demonstrated that the liquid biopsy assay for *MLH1* promoter methylation detection using purely quantitative ddPCR is a simple and highly sensitive procedure that provides reliable methylation detection in ctDNA. The MSRE ddPCR approach can also be applied to other genes of interest where methylation patterns could reveal clinically relevant information for future clinical biomarker and/or companion diagnostic development.

**Supplementary Information:**

The online version contains supplementary material available at 10.1186/s12885-021-08497-x.

## Background

Methylation changes are present in a variety of cancers, and occur early in carcinogenesis, typically repressing the expression of tumor suppressor genes. Acquired promoter hypermethylation often occurs with global hypermethylation of gene promoters known as CpG island methylator phenotype [1]. These epigenetic changes are highly pervasive across a tumor type and can be a very consistent feature of cancer in contrast to mutations, which typically occur at a wide range of sites. *MLH1* promoter hypermethylation is an important event, silencing the *MLH1* gene expression and preventing the formation of MLH1 protein and normal activation of the DNA repair gene. *MLH1* promoter methylation in sporadic tumors causes high levels of microsatellite instability (MSI). Approximately 15% of CRC cases are MSI-High (MSI-H); 3% of which include hereditary polyposis colorectal cancer (HNPCC) or Lynch syndrome and sporadic MSI-H CRC, typically caused by somatic methylation of the *MLH1* gene promoter, making up the remaining 12% of cases [2, 3]. Specifically, within gastric cancer, methylation of CpG islands in the *MLH1* promoter region is frequent resulting in MSI and MLH1 inactivation [4–7]. Emerging evidence has demonstrated that detection of DNA methylation-based biomarkers could be employed for diagnosis, indicating prognosis and predicting response to therapy [8–10]. Therefore, the assessment of *MLH1* promoter methylation status will add essential clinical value across the cancer types.

DNA methylation analysis is not only limited to tissue specimens, but also can be readily extended to be detected in cfDNA/ctDNA, which enables a non-invasive clinical solution. Importantly, the robust and common nature of DNA methylation aberrations in cancer, and the stability of cell-free DNA in body fluids are attractive properties for biomarker development [11–14]. Recently, MSI status could be determined using blood ctDNA test with comparable performance as tissue biopsies-based test [15], potentially providing an alternative pan cancer based approach to monitor patient’s response to therapies.

One of the critical challenges to establishing a DNA methylation assay with clinical usefulness, is identifying the proper region of interest. The investigated region should ideally fulfill the following criteria: first, the region should be unmethylated in normal cases and methylated only in cancer cases; and second, the methylation levels of this region should clearly allow the classification of two distinct populations (for example high versus low or cancer versus healthy). As methylation markers mainly depend on the methylation level of individual CpG sites, the assay sensitivity is limited by the technical difficulties in measuring single-CpG methylation.

Current approaches to methylation analysis depend on bisulfite sequencing, which may cause a degree of DNA degradation and reduce assay reproducibility. Whole-genome bisulfite Sequencing (WGBS) provides complete methylation information, however this approach is not cost-efficient and has no clinical application path. Subsequent optimized approaches such as reduced-representation bisulfite sequencing (RRBS-Seq) and methylated CpG tandems amplification and sequencing (MCTA-seq) aim at capturing CpG-enriched cfDNA fragments, which would lead to loss of some critical DNA methylation sites [16]. Therefore, it is essential to develop a cost-efficient ctDNA methylation analysis approach with improved sensitivity and specificity which could be implemented in a clinical setting. In this study, we developed a methylation sensitive restriction enzyme (MSRE) digested assay to absolutely quantify the *MLH1* promoter methylation in ctDNA using highly sensitive ddPCR in advanced stage CRC samples.

## Materials and methods

### Sample preparation

#### Preparation of sheared methylated/unmethylated DNA

EpiScope® Unmethylated HCT116 DKO gDNA and EpiScope® Methylated HCT116 gDNA reference cell-line DNA samples (catalog #3521, #3522 Takara Bio, Moutainview CA) were sheared to approximately 180 bp by acoustic shearing to mimic fragmented cfDNA using a Covaris Ultrasonicator (Covaris, Woburn MA). DNA size distribution of sheared DNA was tested using Agilent 2100 Bioanalyzer High Sensitivity DNA Kit (catalog #5067–4626, Agilent Technologies, Santa Clara CA) and quantified by Qubit dsDNA High Sensitivity kit (catalog #Q32854, ThermoFisher Scientific, Waltham MA). Fully methylated and unmethylated DNA were mixed to obtain the following ratios of methylation: 0, 10, 25, 50, 75, 100%. Standard curves with known methylation ratios were included in the assay to deduce the methylation level of tumor samples.

#### Sample acquisition and cfDNA extraction

Healthy donor (*n* = 20, median age 44 with range 22–71) and advanced colorectal cancer samples (*n* = 28 (stage IIIB: 21, IIIC: 4, IV:3), median age 63 with range 32–77) used in this study were procured from BioIVT. The written informed consent was received from all subjects used in this study and all samples were collected under IRB approved protocols, as defined by the specimen provider BioIVT. Ethical approval was obtained from BioIVT ethics committee. This study was conducted under the guidelines put forth into the Declaration of Helsinki. No clinical treatment was applied to the CRC patients before blood collection. The healthy controls were recruited as the medical record with colonoscopy confirmation. CRC patients with any immune disease or any other infection (like parasitic infection) which could impact the immune system was excluded. Specifically, blood samples were collected using blood collection tubes containing EDTA (BD Vacutainer®). Plasma isolation was performed within 4 h of blood collection using a double spin protocol: the first centrifugation was performed at room temperature (15 °C to 25 °C) for 10 min at 1600 (± 150) g, then subjected to a second centrifugation step of the plasma supernatant at room temperature (15 °C to 25 °C) for 10 min at 3000 (± 150) g. Plasma nucleic acid was extracted using QIAamp MinElute ccfDNA Kit (catalog #55204, Qiagen, Valencia CA) according to manufacturer’s instructions. Approximately 5 mL EDTA plasma was used for the extraction of cfDNA from normal patient samples. CRC plasma samples ranged in volume from 1 to 4 mL and were brought up to 5 mL each by adding PBS to necessary samples for uniform extraction and to avoid freeze/thaw cycles. Purified nucleic acids were collected in 35 μl elution buffer for both normal samples and CRC samples. Two microliters were used for quantification of extracted cfDNA using Qubit and Bioanalyzer. The donors’ characteristics and cfDNA extraction yield were listed in Supplementary Table 1.

### Methylation sensitive restriction enzyme (MSRE) digestion

MSREs cleave DNA at specific unmethylated-cytosine residues and DNA is then amplified by PCR following digestion. As unmethylated DNA is digested by the MSRE reaction, the only amplification products detected are methylated DNA. Here, a MSRE protocol was optimized for selected *MLH1* promoter region by *HpaII* enzymes. To ensure full digestion of the DNA before PCR amplification to generate accurate data, different *HpaII* enzymes were tested at various concentrations, and with various incubation times. First, two *HpaII* enzymes were identified and tested for complete digestion. Methylated DNA standards and unmethylated DNA standards were treated with each enzyme per manufacturer’s recommendation as well as run in mock digestion reactions where no enzyme was added. Methylated DNA standards and unmethylated DNA standards were tested in qPCR and the calculated percent methylation was determined. The percent methylation was calculated by subtracting the mean Cq values of the MSRE digested templates from the corresponding mock digest (∆Cq). The methylation level was then calculated using the formula;

$$ \mathrm{Methylation}\ \mathrm{level}=\left(2\hat{\mkern6mu} -\Delta  \mathrm{Cq}\right)\ast 100 $$

Ultimately, we determined the ThermoFisher *HpaII* enzyme (catalog #ER0511 Waltham, MA USA) was the better of the two enzymes as the ThermoFisher *HpaII* enzyme had better calculated methylation levels in control samples to the expected methylation levels than *HpaII* NEB#R0171 enzyme (data not shown). Next, we tested different incubation times (2–16 h) and concentrations (2 μL/reaction and 0.5 μL/reaction) to ensure complete digest of the unmethylated samples. Using the same calculations as above, we determined a 3 h incubation with 2 μL of enzyme per reaction was sufficient for complete digestion and extending the incubation time did not significantly improve digestion. Our final MSRE reaction contained: 2 μl 10 × Tango Buffer 3, 2 μl *HpaII* enzyme and 5 ng DNA in total 20 μl final volume. Mock reactions contained 2 μl 10 × Tango Buffer 3 and 2 μl HpaII enzyme. Reactions were incubated at 37 °C for 3 h and subsequently heat inactivated for 20 min at 65 °C. The resulting, 0.25 ng/μL digested DNA was stored at − 20 °C for subsequent analysis.

### Primers and probes design

Beta-Actin (*ACTB*) and Glyceraldehyde 3-phosphate dehydrogenase (*GAPDH*) genes were used as the endogenous reference genes for qPCR and acquired from ThermoFisher (ACTB Taqman Assay ID: Hs01060665_g1, GAPDH Taqman Assay ID: Hs02786624_g1, FAM-MGB). Primers and probe (Forward: 5′-GATGAGGCGGCGACAGA reverse: 3′-AGAAGCAAGATGGAAGTCGAC, probe: 5′-ACCAAATAACGCTGGGTC) of *MLH1* gene promoter were designed to specially amplify the CpG sites and to be located up-stream of exon one (NG_007109.2 region: 4591 -4710), which was interpreted as the promoter regions of the *MLH1* gene using ABI Primer Express version 3.0 (Thermofisher, Waltham MA). The primers and probe were supplied by ABI and optimized for the present study. Briefly, qPCR using FastSYBR Green Master Mix (cat # 4385610, ThermoFisher, Waltham MA) was used to check for nonspecific product formation by dissociation-curve while qPCR using TaqMan Universal Master Mix II (Cat # 4440038, ThermoFisher, Waltham, MA) was used to test the probes and determine the qPCR efficiency.

### Real-time PCR (qPCR)

qPCR was performed in accordance with the Minimum Information for Publication of Quantitative Real-Time PCR Experiments (MIQE) guidelines [17]. The reaction volume was set as 20 μL and each reaction contained 1× Taqman Universal PCR mastermix (cat # 4304437, Life Technologies, Carlsbad CA), 900 nM final concentration forward and reverse primers, 200 nM Probes and 6 ng template DNA. At least one PCR No Template Control (NTC) was run for each assay as controls on all plates. All qPCR analyses were performed in triplicate on a real time PCR system (Model 7500 Fast, Thermo Fisher Scientific®). qPCR thermal cycling conditions were as follows: 95 °C for 10 min, followed by 40 cycles of 95 °C for 15 s, 60 °C for 1 min. The SDS software v2.4 (Life Technologies) was used to calculate the quantification cycle (Cq) value. Invalid PCR results indicated by high cycle threshold (CT) values > 33 were omitted from the analysis.

### Droplet digital PCR (ddPCR)

ddPCR was performed in accordance with the Minimum Information for Publication of Digital Quantitative PCR Experiments (digital MIQE) guidelines (Supplementary Table 2) [18] using Bio-Rad QX200 droplet digital system (Bio-Rad, Hercules, CA, USA). To minimize potential cross-contamination, cfDNA was isolated in a pre-PCR suite inside a PCR hood, separate from instruments used in the amplicon generation process. Following cfDNA isolation, samples were transferred to a designated PCR hood for ddPCR reaction and plate setup. The reaction mixture was comprised of 11 μL of 2× ddPCR Super Mix for Probes (No dUTP) (cat # 1863024, Bio-Rad, Hercules, CA, USA,), 1.1 μL of 18 μM forward primer, 1.1 μL of 18 μM reverse primer, and 1.1 μL of 5 μM probe with FAM label. The remaining 7.7 μL of material was compromised of template DNA and H_2_O based on testing parameters and concentration of sample. Droplets were generated in the QX200 automated droplet generator in our main lab and then transferred to our PCR suite. Our PCR suite physically separates amplicon producing workstreams from the rest of the lab to minimize potential contamination. Reactions were then amplified on an Applied Biosystems VeritiDx thermal cycler using standard ddPCR cycling conditions with an anneal/extension temperature of 55 °C, and finally the plate was read using the QX200 droplet reader (Bio-Rad). The analysis was performed using QuantaSoft software (v1.6.6.0320, Bio-Rad, Pleasanton, CA) as follows. Each plate included a positive control (1 ng human methylated DNA), negative control (1 ng human unmethylated DNA) and no-template controls. Positive droplets, containing amplification products, were discriminated from negative droplets without amplification products by applying a fluorescence amplitude threshold. The threshold was set manually, using the 1D amplitude chart. To assess the specificity of the ddPCR assay, initial experiments were conducted using 100% methylated DNA and 100% unmethylated DNA.

### Data analysis

Values are expressed as mean ± SD, unless otherwise stated. Differences between groups were tested using t test, Mann-Whitney U test, or ANOVA, where appropriate.

Assay performance parameters (sensitivity, specificity and ROC curves) were calculated using the 28 cancer samples and 20 healthy donors. To determine the optimal DNA input amount of the qPCR assay, the standard DNA sample was first prepared as 100 ng μL^− 1^ and then was gradient diluted from 100 ng μL^− 1^ to 1 ng μL^− 1^. For the optimal input amount of the ddPCR assay, the DNA sample was gradient diluted from 6 ng μL^− 1^ to 0.094 ng μL^− 1^. Each assay was conducted in at least three independent runs with four replicates in a run. The limit of detection (LOD) is defined as the lowest detectable percentage of methylation above background and was determined in the ddPCR assay by using the methylation spike-in samples. The linear regression analyses were performed to obtain the correlation coefficient of determination (*R*^2^) and the LOD. The limit of blank (LOB) was determined by the highest number of positive droplets in a No Template Control reaction over the course of several experiments. Receiver operating characteristic (ROC) curves were calculated to establish the cutoffs or threshold methylation level of *MLH1* promoter using the Web-based calculator for ROC curves from online tool http://www.rad.jhmi.edu/jeng/javarad/roc/JROCFITi.html which calculates the cutoff based on the value corresponding with the highest average of sensitivity and specificity. Area under the curve (AUC) indicates the value of the test. Correlation between variables was tested using Spearman’s rank correlation.

## Results

### Assay performance

**Fig. 1:**
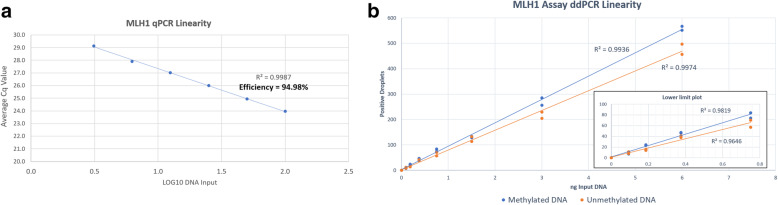
Titration of input amount using synthetic DNA to determine minimum input for qPCR and ddPCR. **a.** Linearity of MLH1 qPCR assay showed very good linearity 0.9987 and efficiency 94.98% with an input range of 100 ng–3.125 ng control DNA. **b.** Comparative analysis of MLH1 promotor methylation using ddPCR demonstrated the ability to detect MLH1 copies in this assay at very low input level (< 1 ng equivalent input). As low as 0.096 ng of MLH1 methylation DNA could be detected by ddPCR

FastSYBR Green PCR revealed a single peak of amplified product following melt curve analysis and TaqMan qPCR efficiency of 94.98% (Fig. 1a) with an input range of 100 ng–3.125 ng. The qPCR assay showed very good linearity > 0.99 indicating good performance of the assay across this range of input. The linearity of the ddPCR assay was determined by titrating the input of sheared methylated control DNA and sheared unmethylated control DNA from 6 ng to 0.096 ng and measuring the number of positive droplets detected. In ddPCR assay, using sheared synthetic DNA, *MLH1* methylation was able to be detected using as low as 0.096 ng of DNA (Fig. 1b).

**Fig. 2:**
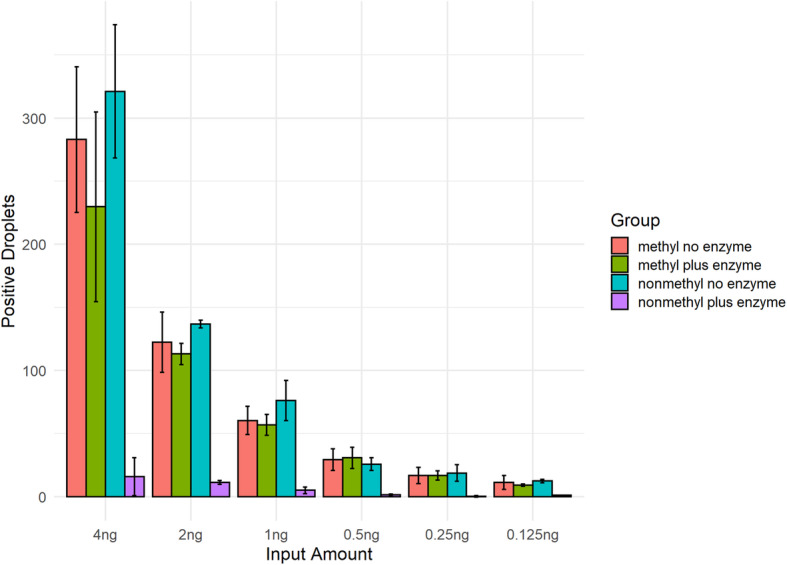
Sample input optimization for *MLH1* promoter methylation ddPCR assay. With 0.125-4 ng input of synthetic DNA, *MLH1* methylation levels detected in the methylated treated, methylated untreated, and unmethylated untreated sample remained fairly constant, the unmethylated enzyme treated sample showed a significant reduction in signal (*P* < 0.001). To achieve the optimal signal/noise ratio and operational/practical advantage, 1 ng was selected as the optimal input for further analysis

To determine the optimal input amount for this assay, a titration of input amounts was tested from 4 ng DNA to 0.125 ng DNA in two-fold dilutions. Four different conditions were tested including methylated DNA treated with *HpaII* enzyme, methylated DNA untreated, unmethylated DNA treated with *HpaII* enzyme, and unmethylated DNA untreated. With 0.125-4 ng input of synthetic DNA, *MLH1* methylation levels detected in the methylated treated, methylated untreated, and unmethylated untreated sample remained fairly constant, the unmethylated enzyme treated sample showed a significant reduction in signal (*P* < 0.001, Fig. 2). To achieve the optimal signal/noise ratio and operational/practical advantage, 1 ng was selected as the optimal input for further analysis.

**Fig. 3:**
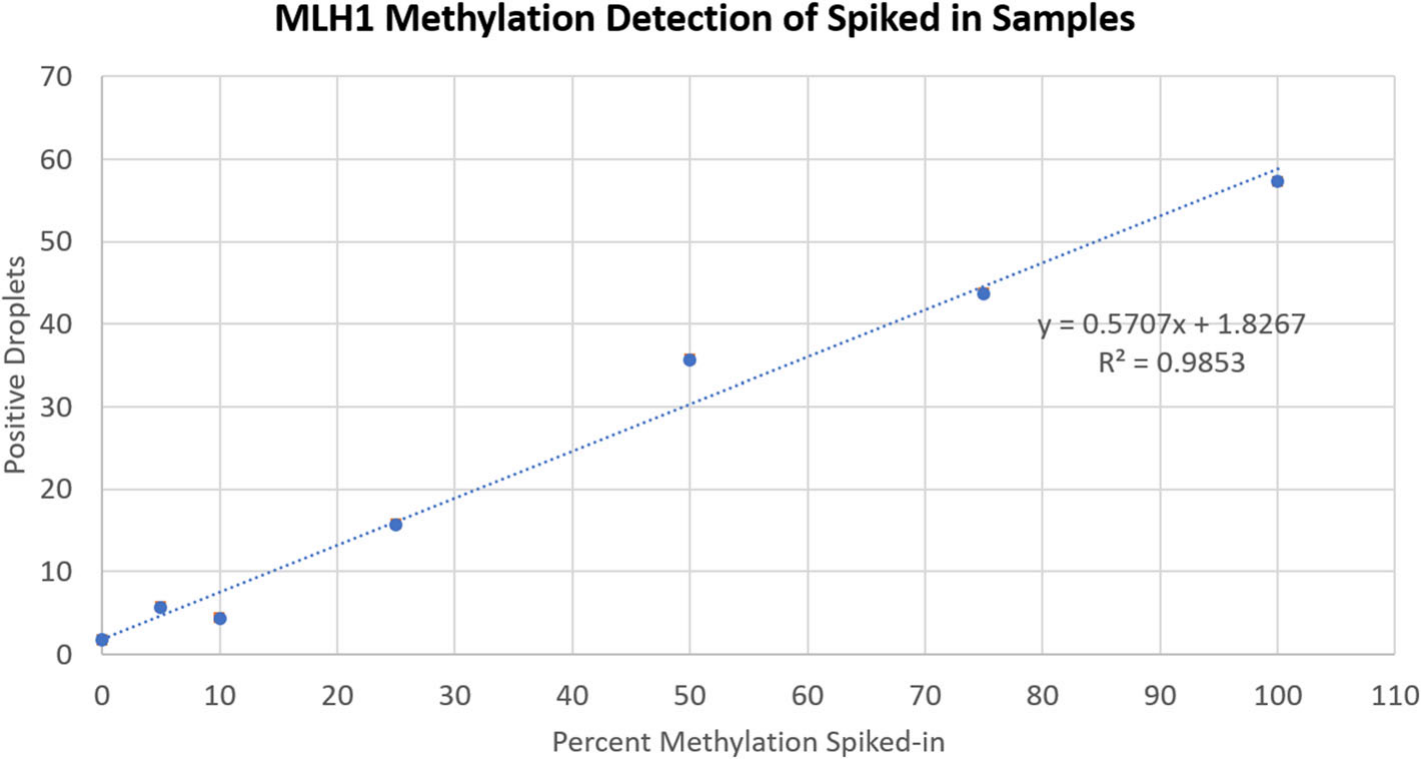
*MLH1* methylation detection of spike in normal plasma sample. Standard curve generated by spiking in 50 ng of DNA with known percentage of methylation into normal plasma, extracting cfDNA from the sample and testing in ddPCR. The normal samples had a measured baseline level of 0 positive droplets. The standard curve gave a R^2^ value of 0.985 and the equation of best can be used to relate positive droplet count to percent methylation

In terms of the percent methylation, the LOD of this assay was determined by spiking different percentages of methylated DNA into normal plasma, extracting cfDNA and then testing 1 ng of DNA in ddPCR to detect *MLH1* methylation levels. While the entire titration showed a good linear relationship overall (R^2^ = 0.985, Fig. 3), levels below 10% methylation displayed a weak linear relationship (R^2^ = 0.427) when examined alone. Therefore, we can consider 10% methylation, or approximately 8 positive droplets, as the LOD for the ddPCR assay. LOB was determined to be 0 positive droplets.

### Testing *MLH1* methylation in healthy controls and CRC patient samples

**Fig. 4:**
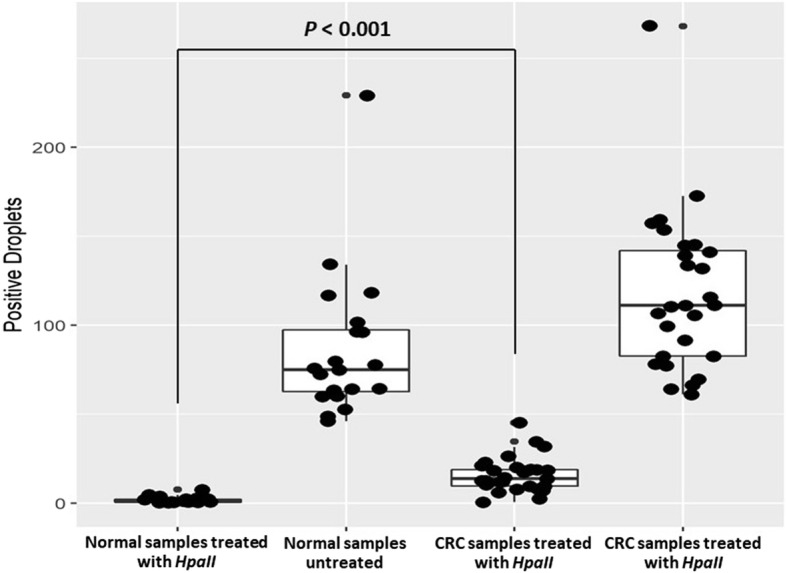
Distribution of detected positive droplets for *MLH1* methylation in Normal and CRC Samples. With 1 ng input cfDNA, 20 normal and 28 CRC samples were treated or untreated with *HpaII* enzyme. In both populations the untreated samples showed higher positive droplets than the treated samples. Compared to normal samples, significant higher methylation levels in the cancer population (*P < 0.001*)

We tested 28 advanced stage CRC cancer samples and 20 normal healthy samples in ddPCR assay to assess the level of *MLH1* methylation in each group (Fig. 4). Except for one sample that failed due to insufficient amount of cfDNA, the rest of samples returned quality ddPCR results. The ddPCR positive droplets for each donor were listed in Supplementary Table 1. First, both groups when untreated showed a significant increase in positive droplets over the treated samples (*P* < 0.001). Normal control samples without enzyme treatment yielded 86.45 ± 41.45 positive droplets for *MLH1* while cancer samples without enzyme treatment yielded an average of 117.39 ± 43.60 positive droplets for *MLH1* (*P* > 0.05). Normal healthy samples treated with *HpaII* enzyme yielded 1.63 ± 1.84 positive droplets indicating that the majority of *MLH1* in normal samples is unmethylated. In contrast the cancer samples treated with *HpaII* enzyme yielded an average of 15.91 ± 9.76 positive droplets suggesting higher methylation levels in the cancer population. A t-test of the cancer and healthy volunteers’ samples was shown to be a statistically significant difference (*P* < 0.001).

**Fig. 5:**
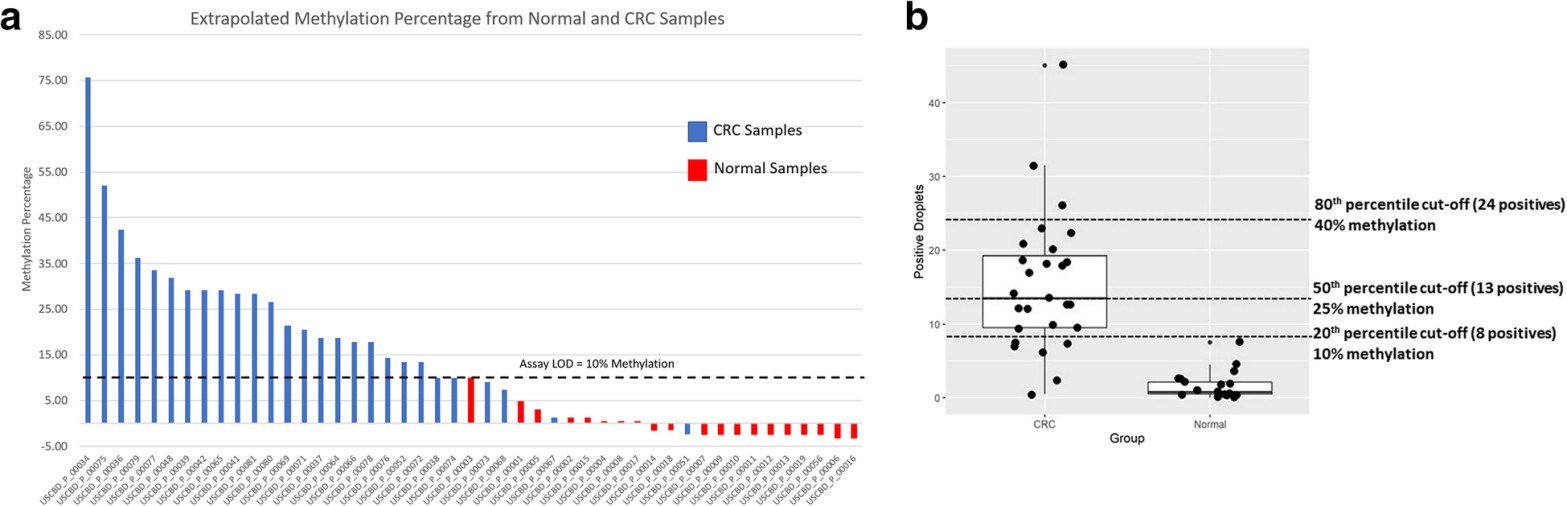
Determining an optimal cut-off to distinguish healthy from CRC patients. a. Based on ddPCR LOD at 10% methylation (approximately 8 positive droplets), all 20 normal patient samples fell below the limit of detection and 21/27 (77.8%) of cancer patients were above this threshold. b. A tiered-based cutoff (20th, 50th, 80th percentile based) to distinguish “methylation high” CRC samples from “methylation low” CRC samples

The data generated here could be used to create a receiver operating characteristic (ROC) curve to differentiate cancer from normal patients with an area under the curve (AUC) of 0.965. The positive droplet count for the normal and CRC patients could then be converted to percent methylation using the equation of the line of best fit from the methylation spike-in standard curve (Fig. 5a). In our cohort all normal patient samples fell below the limit of detection and 21/27 (77.8%) of cancer patients were above this threshold.

**Fig. 6:**
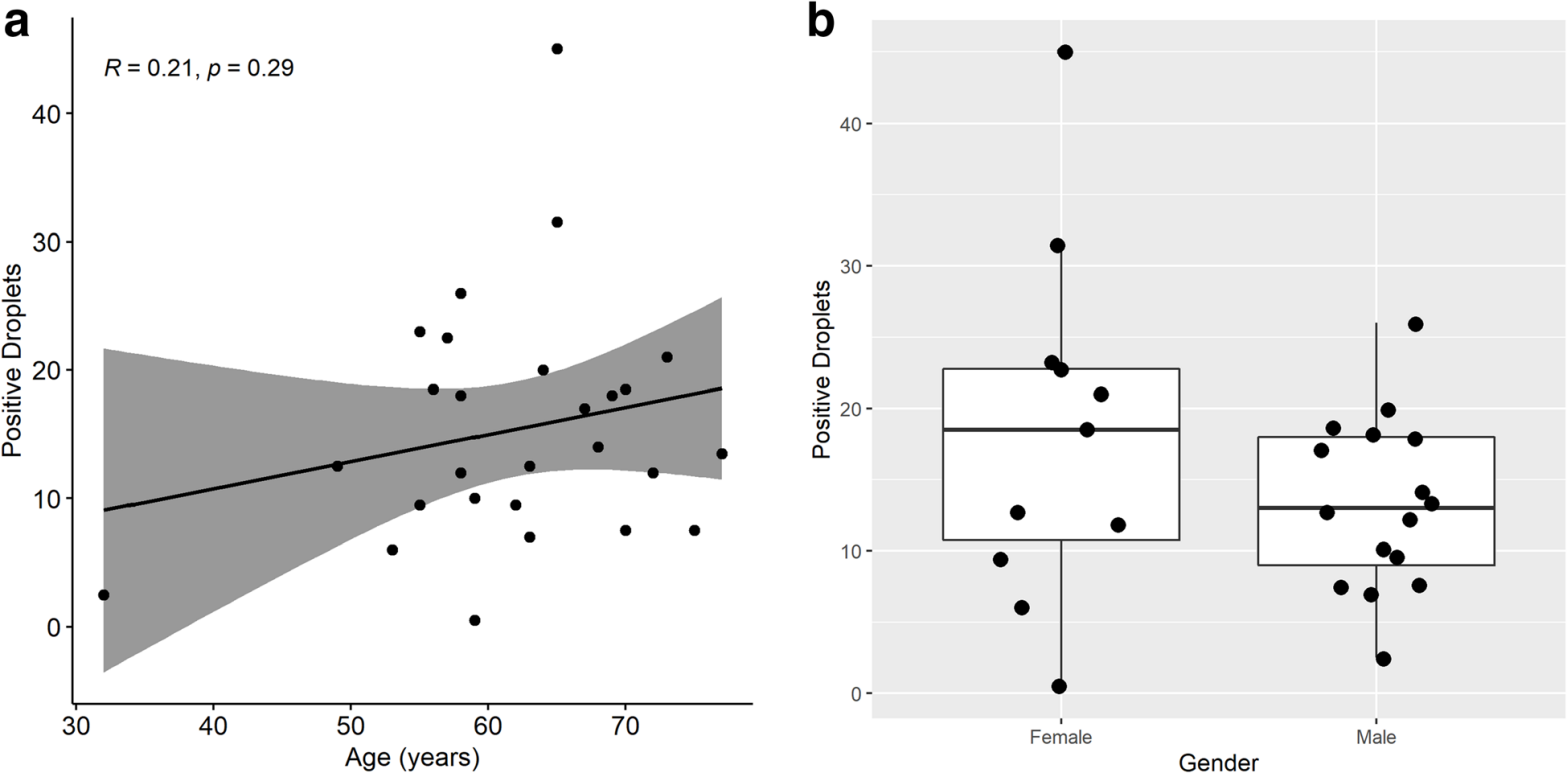
Assessment of the contribution of age (**a**) and gender (**b**) with respect to *MLH1* promoter methylation in CRC plasma samples

By correlating promoter methylation status with age, an increase of methylation levels of *MLH1* with aging observed in CRC samples (*R* = 0.21, *P* = 0.29) (Fig. 6a). Females showed higher *MLH1* promoter methylation levels with respect to males (*P* = 0.23) (Fig. 6b). However, the increase of *MLH1* level with age and female did not reach statistical significance.

### Cut-off value determination strategies

Based on the results of the ddPCR assay, we set up the cut-off values of *MLH1* promoter methylation for discriminating the healthy cases and the malignant cases. Data showed that the optimal sensitivity and specificity was displayed by the assay when using 5 positive droplets as cutoff value (93% sensitivity and 95% specificity, 95% CI: 0.78, 0.98). However, this cut-off was below the reported LOD of the assay. Therefore, the LOD, 8 positive droplets was used as an optimal cut-off to distinguish healthy from CRC patients (78% sensitivity and 100% specificity, 95% CI: 0.45, 0.95 Table 1).

**Table 1:** Summary of sensitivity and specificity calculations at different positive droplets cut off

CUTOFF	1	2	3	4	5	6	7	8	9	10	11	12	13	14	15	16	17	18	19	20
Sensitivity	0.96	0.96	0.93	0.93	**0.93**^**a**^	0.93	0.89	**0.78**^**b**^	0.78	0.70	0.67	0.67	0.52	0.48	0.44	0.44	0.44	0.41	0.26	0.26
Specificity	0.50	0.60	0.85	0.90	**0.95**^**a**^	0.95	0.95	**1.00**^**b**^	1.00	1.00	1.00	1.00	1.00	1.00	1.00	1.00	1.00	1.00	1.00	1.00
ACCURACY	0.77	0.81	0.89	0.91	0.94	0.94	0.91	0.87	0.87	0.83	0.81	0.81	0.72	0.70	0.68	0.68	0.68	0.66	0.57	0.57
PPV	0.72	0.76	0.89	0.93	0.96	0.96	0.96	1.00	1.00	1.00	1.00	1.00	1.00	1.00	1.00	1.00	1.00	1.00	1.00	1.00

^a^ The optimal sensitivity and specificity were displayed at 5 positive droplets as cutoff value (93% sensitivity and 95% specificity, 95%CI: 0.78, 0.98)

^b^ As 5 positive droplets was below the reported LOD, 8 positive droplets were used as an exploratory cut-off to distinguish healthy from CRC patients (78% sensitivity and 100% specificity, 95%CI: 0.45, 0.95)

A tiered-based (20th, 50th, 80th percentile) cutoff was applied to distinguish CRC samples with different methylation level by using the spike-in normal plasma data. A cut-off at the 80th percentile could identify those patients with over *40% MLH1* methylation, those samples falling in between the 50th percentile and 80th would be identified as 25–40% *MLH1* methylation and those samples below the 20th percentile could be considered as ‘low’ *MLH1* methylation (< 10% methylation) (Fig. 5b). However, further clinical analysis of samples would be needed to elucidate the optimal percentiles of the cut-off to ensure a meaningful analysis.

## Discussion

Given the stability of DNA methylation in body fluids, cfDNA methylation markers using plasma samples are a promising matrix for non-invasive clinical biomarker implementation [19]. In addition, global hypomethylation of the human genome paired with CpG islands of hypermethylation, allow for a minimal number of loci to be targeted and yet have sufficient test coverage [20]. These features make cancer-associated DNA methylation changes an attractive source for clinical biomarker discovery [21, 22]. Therefore, targeting DNA methylation changes as cancer biomarkers hold potential for detection and minimal residual disease. However, for such assays to be adopted into a clinical setting, high sensitivity and specificity need to be demonstrated. In many cases it is crucial for the assay to detect only a few cancer specific DNA methylation changes in a large background of normal DNA methylation patterns. We developed a MSRE digested ddPCR assay to quantitate the methylation of *MLH1* promoter in cfDNA for detection of low concentrations of methylated DNA fragments in advanced stage CRC.

While many other ctDNA methylation biomarkers for cancer have been reported [9, 23–25], these mainly depend on the methylation level of individual CpG sites, which limits the detection sensitivity. Until now, only one blood-based assay that detects *SEPT9* gene methylation was approved by the U.S. Food and Drug Administration for CRC screening with a sensitivity of ~ 70% and a specificity of ~ 80% or above [26]. Our assay tests the methylation level of the *MLH1* promoter region and offer a cost efficient, clinical applicable alternative approach compared to bisulfite-treatment approaches. A MSRE assay followed by ddPCR achieves absolute quantification of the degree of methylation of the DNA with minimal (1 ng) cfDNA input and the assay does not rely on a standard calibration curve. Using the control DNA samples, we have proven that the LOD of the ddPCR assay is supersensitive, with 25-fold improvement compared to the conventional qPCR-based assay. Based on the results of the ddPCR assay, ROC curve produced from the data gave an AUC of 0.965 indicating the assay does a good job of differentiating normal samples from cancer samples.

Moreover, we applied comprehensive approaches to determine the cut-off value for potential clinical applications. First, a statistical cut-off of 8 positive droplets with optimal sensitivity and specificity (78% sensitivity and 100% specificity) of the assay for discriminating healthy volunteers from CRC patients was determined. With the cut-off of 8 positive droplets, 21/27 (77.8%) of cancer patients were above this threshold. In the early assay development, only limited number of advanced stage CRC patient’s plasma samples were included for analysis which may lead to higher positivity rate. A recent study observed the stage-stratified sensitivity of selected 3 CRC-specific DNA methylation markers in bisulfite-converted cfDNA [27]. Therefore, selection of different stage of CRC patients and matched tissue samples are necessary to improve the assay performance of this approach. The hypothetical cutoff and clinical significance will be validated in an independent cohort in the future study. Second, a tiered cut-off was applied to distinguish CRC samples with different methylation levels (20, 50, 80%). Coppede et al. demonstrated that *MLH1* promoter level in CRC tissue is highly correlated with MLH1 protein expression using immunostaining [28]. Their results showed that in the vast majority of cases (80%) if promoter methylation was present, MLH1 immunostaining was negative, and, in contrast, in 93% of cases with 0% promoter methylation MLH1 immunostaining was positive. In the 20% of cases with both positive MLH1 promoter methylation and positive immunostaining, the overall promoter methylation level was low [28]. Therefore, it is reasonable to speculate that the tiered *MLH1* methylation level is linked to MLH1 protein expression and could be further developed as a complementary assay of IHC tests for mismatch repair deficient (dMMR) to identify the MSI-H or dMMR tumor status.

Our analysis revealed that *MLH1* promoter methylation of ctDNA in plasma tended to increase with age, as well as gender differences with females showing higher hMLH1 promoter methylation than males in CRC patients (Fig. 6). Although there is no statistical significance due to insufficient power, our results showed the similar trend observed in CRC tissue as previous reported [28]. These findings provide evidence that *MLH1* promoter methylation signals could be captured in plasma. Recent publications have established an association between the epigenetic alterations found in primary tumor specimens and in plasma, suggesting epigenetic biomarkers from liquid biopsy can be implemented as surrogate tumor biomarkers [29–33]. However, the amount of ctDNA in plasma can be related to tumor stage, proximity of the tumor to the bloodstream, metastasis, and other confounders such as exercise and dietFor these reasons, the variability of the total cfDNA person-to-person may affect the detection sensitivity. Further studies will be needed to elucidate how these factors influence the analytical detection of *MLH1* promoter methylation in plasma samples.

The tissue based pooled frequency of *MLH1* promoter methylation in CRC is reported as ~ 18% in sporadic CRC [34, 35]. DNA methylation of *MLH1* has been found in more than 80% of sporadic CRC with MSI and predict the mutation load. Thus, the assessment of *MLH1* promoter methylation status will add essential diagnostic and prognostic information with clinical relevance. In our study, a theoretical cut-off methylation levels based on real-world data was set at ~ 24 positive ddPCR droplets (equivalent to 40% methylation in Fig. 5b) in CRC plasma samples. As the current study is relatively small, proper validation using a different cohort with a larger sample size is crucial. Comprehensive and fit-for-purpose cut-off determination strategies need to be considered to fulfill the different project goals and clinical application specific requirements.

## Conclusion

The developed liquid biopsy assay for detecting *MLH1* promoter methylation by ddPCR is a convenient and cost-efficient approach which could be easily transferred to a clinical setting. The simple MSRE ddPCR procedure and high sensitivity of the assay provides a reliable ctDNA methylation biomarker assay to reflect single or multiple DNA methylation changes originating in tumor cells. Furthermore, this approach can be applied to other genes of interest, or cancer indications, where methylation patterns could reveal crucial clinically relevant information for future clinical biomarker and/or companion diagnostic development and implementation.

## Supplementary Information

**Additional file 1.**

**Additional file 2.**

## Data Availability

The datasets used and/or analysed during the current study are available from the corresponding author on reasonable request.
